# Estrogen Signaling in Lung Cancer: An Opportunity for Novel Therapy

**DOI:** 10.3390/cancers4040969

**Published:** 2012-09-25

**Authors:** Christina S. Baik, Keith D. Eaton

**Affiliations:** 1 Division of Medical Oncology, Department of Medicine, University of Washington, Seattle, WA 98109, USA; E-Mail: kdeaton@u.washington.edu; 2 Fred Hutchinson Cancer Research Center, Seattle, WA 98109, USA

**Keywords:** lung cancer, estrogen, progesterone, aromatase, hormone receptors

## Abstract

Lung cancer is the leading cause of cancer death in U.S. and represents a major public health burden. Epidemiologic data have suggested that lung cancer in women may possess different biological characteristics compared to men, as evidenced by a higher proportion of never-smokers among women with lung cancer. Emerging data indicate that female hormones such as estrogen and progesterone play a significant role in lung carcinogenesis. It has been reported that estrogen and progesterone receptors are expressed in lung cancer cell lines as well as in patient-derived tumors. Hormone related risk factors such as hormone replacement therapy have been implicated in lung carcinogenesis and several preclinical studies show activity of anti-estrogen therapy in lung cancer. In this review, we summarize the emerging evidence for the role of reproductive hormones in lung cancer and implications for lung cancer therapy.

## 1. Introduction

Lung cancer represents a major public health burden in the U.S. with an estimated 221,000 new cases and 157,000 deaths annually [1]. Lung cancer mortality began to decrease in 1990s for men, while it increased steadily for women until the mid-2000s [2]. Lung cancer mortality in women began to decrease for the first time during years 2003–2007, likely reflecting the later uptake of cigarette smoking by women and the changes in smoking prevalence in the last several decades [3]. In addition to the differences in smoking behavior, epidemiologic data have suggested that lung cancer in women may possess different biological characteristics compared to men, as evidenced by the higher proportion of never-smokers among women with lung cancer. For instance, approximately 15% of lung cancer develops in never-smoking women compared to 5–10% in men in North America and approximately 20% of lung cancer in men and 50% in women are attributed to factors other than smoking globally [4,5]. Also, lung cancer incidence in never-smoking women has been reported to be higher, with an incidence rate of 14.4–20.8 per 100,000 person-years, compared to 4.8–13.7 in men in a multi-cohort study [6]. Additionally, it is now well recognized that the epidermal growth factor receptor (EGFR) mutation positive lung cancers are more common in women and never-smokers [7].

The above findings have led researchers to investigate whether female hormones such as estrogen and progesterone could play a significant role in lung carcinogenesis. Studies show that estrogen receptors (ER), especially the ERβ subtype, and progesterone receptors (PR) are expressed in human lung cancer cell lines as well as in patient-derived tumors [8]. Additionally, there is evidence from preclinical studies that β-estradiol is tumor promoting in lung cancer-derived cell lines and the administration of an ER antagonist decreases cellular proliferation in NSCLC cell lines and in tumor xenograft models [8,9]. These findings support the hypothesis that the estrogen pathway and other related hormonal pathways likely play an important role in lung carcinogenesis and active research is ongoing to address this question. In this article, we review the recent advances in our understanding of the role of estrogen and other associated hormones in lung carcinogenesis and propose future steps needed to bring the knowledge closer to clinic.

## 2. Estrogen and Lung Cancer

The importance of the estrogen pathway in various physiologic functions and in carcinogenesis has been extensively investigated over the past decades, especially in the context of breast carcinogenesis. Estrogen receptors are well known members of the nuclear steroid receptor superfamily; the two main types of ER, ERα and ERβ, exert their influence on cellular functions by initially binding to their ligands which are the naturally occurring estrogens: estrone, 17β-estradiol, and estriol [10,11]. Estradiol is the primary reproductive hormone that is mainly synthesized in the ovary under the stimulation of follicular stimulating hormone and luteinizing hormone, while estrone and estriol are mostly synthesized in the liver from estradiol [12]. The two ER subtypes, ERα and ERβ, are products of separate genes, ESR1 and ESR2 respectively, with ESR1 located on chromosome 6 and ESR2 on chromosome 14 [13,14]. ERα was first cloned in 1986 and its expression is highest in breast, ovarian and endometrial tissues [10,12]. The discovery of ERβ has been more recent, with human ERβ being identified in 1996 [15] with a wide tissue distribution including the lung, kidney, colon, bone, brain, endothelial cells, prostate, testes and ovaries [12,14]. Both receptors contain functional domains, AF1 and AF2, which are involved nuclear signaling. While AF1 is a ligand-independent transcriptional activation domain located near the amino-terminus, AF2 is located near the carboxy-terminus and acts as a ligand-dependent activation domain of the receptor. The centrally located DNA binding domain is highly conserved with amino acid sequence homology of 95% between the two receptor types while the ligand binding domain, AF2, shows 55% homology between the two receptors resulting in varying affinities in the two ER subtypes [15]. The two subtypes exhibit similar affinity to 17β-estradiol, but ERα binds to 17α-estradiol and estrone with higher affinity, while ERβ has higher affinity to estriol [12,15]. Upon binding of a ligand to AF2, receptors form either homo- or heterodimers and bind to the estrogen responsive element (ERE) within the promoter of estrogen responsive genes resulting in regulation of target gene transcription [16]. While both ERα and ERβ contain potent AF2, the AF1 domain in ERβ is truncated and less active at inducing transcriptional activation, thus ERβ is more dependent on AF2 for its transcriptional activity [17].

### 2.1. Estrogen in Normal Lung Physiology

The importance of biological functions of estrogen and its receptors in reproductive organs has been understood for several decades. Currently there is increasing recognition that the biological activity of estrogens is not limited to reproductive organs but that it has important functions in organ systems that are not traditionally considered to be under hormonal influence. In the lung, ERβ is highly expressed in pneumocytes and in bronchial epithelial cells and is required for the maintenance of the extracellular matrix of the lung. The loss of ERβ in knockout mice has been shown to result in the development of abnormal lung structure and systemic hypoxia [18]. In a study comparing lung tissues of ERβ wildtype mice and mice with inactivated ERβ gene, the lung tissues of ERβ null mice were found to have decreased number of alveoli, decreased levels of surfactant, platelet derived growth factor A, and granulocyte-macrophage colony stimulating factor [19]. Other studies of ER deficient mice have shown that ERα mediates the determination of alveolar number and surface area while ERβ affects the lung elastic tissue recoil [20].

### 2.2. Estrogen Receptor Expression in Lung Cancer

Given the increased understanding of estrogenic influence in normal lung physiology and the epidemiologic observation of higher proportion of never-smokers in female lung cancers, there has been increased interest in exploring the role of estrogen in lung cancer. Examination of estrogen receptors on lung tumor tissues has been performed since the 1980s with one of the earliest reports by Beattie *et al.* who examined the incidence of steroidal hormone receptor expression in 55 resected non-small cell lung cancer (NSCLC) tumor specimens via a sucrose gradient analysis method. The investigators of this study reported a 30% expression of ER in normal lung specimens and a similar expression in squamous and adenocarcinoma tumors [21]. Another study employing a similar sucrose gradient method reported 18% positive ER expression in 17 resected NSCLC specimens [22]. Despite the small number of tumor specimens, these studies provided early evidence for ER expression on lung tumors and raised the hypothesis that the receptors could play a significant function in lung carcinogenesis. Since the initial published studies in the 1980s, there have been a number of subsequent studies evaluating hormone receptor expression, with the majority of studies using an immunohistochemical methodology. This allowed the determination of the subtype of ER, ERα or ERβ, as well as the localization of expression to the nucleus or cytoplasm.

However, the presence of ER, especially ERα in human lung tumors, has been controversial for many years, with some studies showing high rates of ERα positivity, while others reporting the opposite [11]. Studies have reported both nuclear and cytoplasmic expression of ERα ranging from 0–80% and ERβ ranging 9–98% [13,23,24,25,26,27,28,29,30,31,32,33,34,35,36,37,38,39,40,41,42] (Table 1). This observed inconsistency is likely due to the heterogeneity in methodologies including the specific antibodies used for immunohistochemical staining as well as heterogeneous definitions of positivity. It has been reported that lung tumors express mostly splice variants of ERα, while they express both full-length and splice variants of ERβ [8]. A comparison of ER expression of a breast cancer cell line (MCF-7) with lung cancer cell lines (H23, 91T) demonstrated that while the full-length ERα was often observed in the breast tumor cell lines, lung cancer cell lines exhibited a small amount of full-length receptors and mostly expressed products of splice variants. Both full-length as well as splice variants of ERβ were observed in lung cancer cell lines [8]. Many of the initial studies reported ERα expression in lung tumors using antibodies commonly used in breast cancer studies, thus false negative findings may have resulted if the antibodies targeted epitopes that are not present in splice variants found in lung tumors. To address this issue, a study examined ERα expression in lung adenocarcinoma tumor samples using three anti-ERα antibodies, mouse monoclonal 1D5 and 6F11 and rabbit monoclonal SP1. The three antibodies were used to target three specific domains of ERα with 1D5 recognizing the amino-terminal domain, 6F11 targeting the full-length receptor, and SP-1 targeting the carboxy-terminal domain. Interestingly, the reported nuclear ERα expression varied by antibody used with 7.6% expression with 1D5, 14.1% with 6F11 and 27% with SP-1, suggesting that reactivity was higher with an antibody that targets the carboxy-terminal end of the receptor [43]. Similar methodology was used in an independent study which reported a 56–80% nuclear ERα expression with 6F11, but no ER expression with the 1D5 antibody [37]. However, there are studies which contradict these findings. For instance, a study examining 4 commercially available ERα antibodies reported varying expression ranging 7–54% in the nucleus and 0–42% in the cytoplasm of lung adenocarcinoma specimens [24]. In this study, the antibody HC20 which targets the carboxy-end resulted in a similar expression as the antibody 1D5 which targets the amino-end, indicating that variability in the literature may not be solely due to the variability in carboxy- *versus* amino-end targeting. Nonetheless, in the overall review of published studies, those using amino-terminal targeting antibodies report lower expression ranging from 0–40% with several studies reporting no expression [29,37,38,39] compared to studies which use carboxy-terminal targeting antibodies reporting expression of 36–79% [23,24,26,33,36,43,44]. Expression with 6F11, which targets the full-length receptor is more variable ranging from 0% to 80% [29,33,34,35,37] (Table 1).

**Table 1:** Selected review of estrogen and progesterone receptor expression in NSCLC.

ERα		ERβ		PR		n	Ref
Antibody	%	Antibody	%	Antibody	%		
HC20	49	H150	49	--	--	447	[23]
HC20	36	--	--	SP2	45	316	[26]
HC20	52	--	--	--	--	122	[36]
HC20	79	MCA1974S	97	MAB429	91	183	[44]
HC20	0–48	H150	54–98	SP2	58–70	317	[24]
1D5	17–40	14C8	16–48				
6F11	0–45						
Biogenex	0	BioGenex	46		--	301	[28]
1D5	19	--	--	AR441	8	64	[32]
1D5	0	--	--	hPRa3	0	248	[39]
--	0	14C8	50	Ncl-PgR	0	127	[45]
--	--	14C8	86	--	--	262	[42]
1D5	0	Cosmobio	67	--	--	30	[38]
--	0	14C8	50	--	0	127	[31]
1D5	14	PPG5/10	9	PgR, Dako	12	109	[27]
1D5	9	Biogenex	38	PgR, Dako	11	100	[25]
1D5	0	--	--	--	0	45	[37]
6F11	55						
1D5	0	MCA1974S	61	--	--	278	[29]
6F11	0						
6F11	78	--	76	sc543	12	58	[33]
6F11	<1	14C8	71	MAB429	13	105	[34]
6F11	54	MS-ERβ13-PX1	90	--	--	59	[35]
6F11	38	14C8	33	MAB429	47	228	[46]

--: data not available.

The expression of ERβ appears to be more consistent ranging from 9% to 98% with most studies reporting greater than 30% expression [23,24,25,27,28,29,30,31,33,34,35,38,41,42]. Lung tumors express both full-length and splice variants of ERβ thus reducing variability with the use of antibodies that target different isoforms of the protein. ERβ expression was evaluated using antibodies targeting the amino- (14C8) and carboxy-end (GC17/385P) of ERβ [31]. This study reported 49% expression using the amino-end targeting antibody and 48–51% with the antibody targeting the carboxy-end demonstrating that expression of ERβ appears to be consistent regardless of the targeted region of the protein.

Another source of variability in the reported ER expression is the heterogeneity in the definition of a positive result (Table 2). Most studies use a scoring system which integrates the immunohistochemical staining intensity and the percentage of positive tumor cells. Several studies use the Allred score commonly used in breast cancer [47] which is the sum of the score of the proportion of positive cells and the intensity score generating a score ranging 0–8 [23,44]. A few studies use a pre-specified thresholds while others use the median as the cut off value [24,29,31,45]. The correct definition of positivity in lung cancer is currently not known and this will depend on the clinical significance of ER expression. A published guideline for breast cancer from American Society of Clinical Oncology (ASCO) recommends a cutoff of a minimum 1% of positive tumor cells for a tumor sample to be deemed positive given that levels as low as 1% can predict treatment response to endocrine therapy in breast cancer [47]. A clinically relevant definition of positivity in lung cancer will be determined by its ability to serve as a prospective prognostic or predictive biomarker in future studies.

**Table 2:** Definitions of ER positivity in NSCLC used in literature.

Scoring method	Positivity	Ref
Scoring formula = [(3x) + (2y) + (1z)]/100 where x, y, and z are % staining at intensity 3, 2 and 1, respectively	High *versus* low expression defined by dichotomizing at the median value	[45]
Staining intensity: 0 (none), 1 (weak), 2 (moderate), 3 (strong)		
Percentage of cells staining at each intensity level (0–100%)		
Proportion and intensity score added together to obtain a total score ranging 0–8 (Allred score)	Negative expression: 0	[23,44]
Proportional score for positive staining:	Weak: 2–4	
0 (none), 1 (≤1% positive tumor cells), 2 (2–10%), 3 (11–33%), 4 (34–66%), 5 (>67%)	Strong: 5–8	
Intensity score: 0 (none), 1 (weak), 2(intermediate), 3 (strong)		
Percentage of staining cells: (<10%, 11–50%, 51–100%)	>10% tumor cells with at least 1+ staining	[29]
Staining intensity: 1 (weak), 2 (moderate), 3 (strong)		
Scoring formula = intensity × percentage of positive cells (overall IHC score 0–300)	IHC score equivalent or greater than the median	[31]
Intensity (0, 1, 2, 3+)		
Percentage of positive cells (0–100%)		
Scoring formula = intensity × percentage of positive cells (overall IHC score 0–300)	Any expression score >0	[24]
Intensity (0, 1, 2, 3+)		
Percentage of positive cells (0–100%)		

The above methodological heterogeneity presents significant challenges when interpreting lung cancer estrogen receptor expression results in the literature. Nonetheless, the above studies provide strong evidence for presence of estrogen receptors in NSCLC and emphasize the importance of consistency in methodology, especially the use of validated antibodies and a consistent definition of positivity in future studies.

### 2.3. Estrogen Receptor and EGFR Mutation

Given the observation that EGFR mutation lung adenocarcinoma is more commonly found in women compared to men, several studies have evaluated the association between ER expression and epidermal growth factor receptor (EGFR) mutation. A study from Japan evaluated 447 resected adenocarcinoma specimens for ER expression and its association with EGFR mutations. This study reported an overall expression of 49.4% cytoplasmic ERα and 48.5% nuclear ERβ. This study also reported that EGFR mutation status was associated with higher nuclear expression of ERβ, with 67% of EGFR mutation positive tumors strongly expressing ERβ *versus* 37% in EGFR wildtype tumors. There was no difference in ERα expression distribution [23]. Another independent study evaluated 317 NSCLC samples and reported higher ERα and ERβ expression associated with EGFR mutation positive samples with 82% of EGFR mutant tumors harboring ERβ nuclear expression compared to 48% in wildtype tumors [24]. This data suggests that the estrogen pathway may play an important role in EGFR mutation positive lung cancers and warrants further investigation into the mechanism of interplay between ER and EGFR pathways.

### 2.4. Mechanism of Estrogen in Lung Carcinogenesis

Studies have demonstrated that estrogen receptors are not only expressed in lung cancer but play important biologic functions in the carcinogenic process. Similarly to what is observed in breast tumor cell lines, cell line experiments have shown that administration of β-estradiol stimulates growth of lung tumor cells [8]. Consistent results have been observed in *in vivo* experiments in which estradiol administration stimulates lung tumor growth in mouse models. This was demonstrated by Hammoud *et al.* who examined the effect of estradiol in lung adenocarcinoma mouse models expressing the oncogenic form of K-ras with concurrent deletion of Tp53 [48]. Results showed that tumor volumes were decreased by ovariectomy in females and that estradiol increased tumor counts and volume in the ovariectomized mice. It has been hypothesized that the overall mechanism of the estrogen pathway in lung cancer is likely similar to what is known in breast carcinogenesis, although there are important differences with estrogen playing a major role in the initiation of the malignant process in breast cancer while in lung cancer, estrogen likely plays a greater role in tumor progression and metastatic spread [49,50,51].

The literature indicates that estrogen exerts its influence in lung carcinogenesis by both genomic and non-genomic signaling. In the classical model of genomic signaling, ligands bind to the nuclear ER resulting in homo- or heterodimers. The ligand-receptor complex then binds to the estrogen response elements (ERE) within the promoter of estrogen responsive genes and initiate transcription of target genes [52]. The classical model of genomic estrogen signaling has been demonstrated in lung cancer cell line experiments. In an ERβ expressing lung cancer cell line (RERF-LC-OK) transfected with an ERE-luciferase reporter plasmid, the addition of 17β-estradiol resulted in increased transcriptional activity and an anti-estrogen, ICI 182,780, inhibited the ER mediated ERE-transcription [38]. Another independent study also demonstrated that β-estradiol induced transcription of an ERE-luciferase construct in lung tumor cell lines [8].

Non-genomic signaling by ER can occur via several mechanisms including activation of epidermal growth factor receptor (EGFR) and mitogen-activated protein kinase 1 (MAPK1) pathways by membrane associated ERs [50,53]. Evidence for ER mediated non-genomic signaling in lung cancer has been reported in several *in vitro* studies which demonstrate that that membrane ERs are co-localized with EGFR and 17β-estradiol administration results in rapid phosphorylation of the MAPK pathway in lung cancer cell lines, thus indicating membrane signaling in NSCLC by ER [9,54,55,56]. There is evidence of a feedback mechanism between ER and EGFR in which upregulation of EGFR protein expression is observed in response to anti-estrogens and decreased ERβ expression is seen in response to EGF administration [9]. Emerging data also indicate that mitochondrial ERβ may play a role in inhibiting apoptosis by physically interacting with the pro-apoptotic protein Bad and disrupting the Bad-Bcl-XL and Bad-Bcl2 interactions [57].

### 2.5. Other Associated Hormones in Lung Cancer

Although there has been much emphasis on the estrogen pathway in recent literature, there are other hormonal receptors and enzymes associated with the estrogen pathway which likely play important functions in lung carcinogenesis. Several studies have reported progesterone receptor (PR) expression in lung tumors ranging from 8% to 63% [24,25,26,27,32,33,34,46] although there are studies reporting no expression [31,37,39] (Table 1). The biological effect of PR on lung tumorigenesis has been inconsistent in the literature with reports of progesterone resulting in growth inhibition of PR positive lung tumors [46] as well as improved survival of lung cancer mouse models by anti-progestin treatment [58]. While there may be a potential direct effect of progesterone on PR expressing tumors, there is increasing evidence that progesterone exerts its influence by promoting angiogenesis. In an *in vitro* experiment of embryonic lung tissue, administration of estrogen and progesterone resulted in increased vascular endothelial growth factor (VEGF) expression and this effect was abolished with anti-estrogen and anti-progestin therapy [59]. Similar results have been observed in independent experiments where treatment of lung cancer cell lines with progesterone or 17β-estradiol resulted in increased VEGF secretion which was blocked by administration of an anti-estrogen or anti-progestin therapy. Moreover, this study also demonstrated increased endothelial cell proliferation with progesterone administration suggesting a biologically meaningful effect by progesterone on tumor angiogenesis [60]. The above data provide convincing evidence that progesterone plays an important role in tumor angiogenesis and anti-progesterone therapy may be a promising treatment strategy.

Emerging data also indicate that intratumoral aromatase likely plays an important function in lung cancer as well. Aromatase is a cytochrome P-450 enzyme that converts androstenedione to estrone and testosterone to estradiol [61]. Several studies have reported 64–86% aromatase expression in lung tumors [26,34,35,62] and the expression has been associated with more aggressive tumor characteristics [35]. In an analysis of NSCLC resected tumors, concentration of estradiol was higher intratumorally compared to normal lung tissues from same patients, and intratumoral estradiol concentration was associated with aromatase mRNA expression. The study also found that intratumoral estradiol concentration correlated with larger tumor size and higher Ki-67 in ER expressing tumors but not in tumors that did not express ER. *In vitro* experiments from the same study showed that estradiol induced cell proliferation in an ER expressing NSCLC cell line which was inhibited by an anti-estrogen and also by an aromatase inhibitor [35]. These studies demonstrate that there is interplay between estrogen receptors, intratumoral aromatase, and intratumoral estradiol influencing lung tumorigenesis.

## 3. Prognostic Implications of Estrogen Receptor Expression in Lung Cancer

While there is growing literature reporting the presence of hormonal receptor expression in lung cancer and results from preclinical models demonstrating that they play significant functions in lung tumorigenesis, this information has not yet been translated into clinical practice. It is not yet clear whether hormone receptor status could provide reliable prognostic information or guide therapies. Several studies have investigated the potential prognostic significance of ER and PR expression; however, results have been inconsistent, with a number of studies reporting ER to be associated with good prognosis [25,28,30,31,33,34] while others report the opposite [24,27,32,36,44,45]. Other studies have reported ERβ to be associated with good prognosis in men but poor prognosis in women [29,41]. The observed inconsistency is likely due to the wide heterogeneity in the methodology used, for hormone receptor assessment as well as in the analysis of clinical data, such as adjustment for known prognostic factors. Interestingly, results with aromatase expression are more consistent, with several studies reporting aromatase to be associated with poor prognosis [44,45,63]. A study examined NSCLC tumor samples from 377 patients for ERα, ERβ, and aromatase expression and associated the expression with survival outcomes of these patients. The results revealed that neither ERα nor ERβ alone or combined expression predicted disease specific survival but combined ERβ and aromatase expression was predictive with patients whose tumors harbored high expression of aromatase and ERβ having a lower survival when compared with those with high aromatase and low ERβ expression, especially in women who were 65 years or older [45]. Combined aromatase and ERβ expression remained a significant predictor of survival after adjustment of clinically significant covariates including tumor grade, stage, and age, and this was observed in both men and women. Interestingly, combined ERα and aromatase expression did not predict survival, suggesting that ERβ may be the more biologically active ER subtype in lung tumorigenesis. These findings are consistent with an independent observation that intratumoral estradiol concentration is correlated with larger tumor size and higher Ki-67 in ER expressing tumors but not in tumors that do not express ER [35]. The above studies suggest that ER expression alone likely will not provide sufficient prognostic information and indicates that the combined analysis of both aromatase and ER expression is a promising approach that should be validated in independent studies.

## 4. Hormone Receptors as Predictive Markers of Lung Cancer Therapy

There currently is no clinically proven hormonal therapy for lung cancer. However, there are several estrogen modulating treatments that are widely used in breast cancer which could be explored in lung cancer therapy (Table 3). Several preclinical studies have shown that hormonal therapy can reduce lung tumor growth [8,48] and this was demonstrated by an *in vivo* experiment where immunocompromised xenografts with ER expressing lung tumors were treated with estradiol, estradiol with fulvestrant (an estrogen receptor antagonist), or fulvestrant alone. This study showed that treatment with estradiol resulted in tumor growth while treatment with fulvestrant resulted in approximately 40% tumor growth inhibition [8]. Subsequent studies have evaluated the addition of an EGFR targeting compound to fulvestrant with the goal of blocking both the ER and EGFR pathways [9,61,64,65,66]. A study of immunocompromised mice with ER and EGFR expressing lung tumors demonstrated that estrogen significantly stimulated tumor growth which was inhibited by fulvestrant and gefitinib (an EGFR tyrosine kinase inhibitor). Moreover, the combination of gefitinib and fulvestrant resulted in greater tumor reduction compared with either treatment alone [9]. 

**Table 3:** Endocrine therapy in metastatic breast cancer.

Name	Class	Mechanism	Route of administration	Studies in NSCLC
Exemestane	Aromatase inhibitor, steroidal	Inhibits aromatase, irreversible	Oral	Tumor reduction in lung cancer xenografts [61]
Anastrozole	Aromatase inhibitor, nonsteroidal	Inhibits aromatase, reversible	Oral	Ongoing phase II clinical trial [67]
Letrozole	Aromatase inhibitor, nonsteroidal	Inhibits aromatase, revesible	Oral	Decreased cell proliferation in ER expressing cell lines [35]
Letrozole	Aromatase inhibitor, nonsteroidal	Inhibits aromatase, revesible	Oral	Decreased cell proliferation in ER expressing cell lines [35]
Fulvestrant	Estrogen receptor antagonist	Competitively binds to estrogen receptors, antagonistic effect only	Intramuscular	Decreased cell proliferation *in vitro* and tumor reduction in lung cancer xenografts [8,61,65], ongoing phase II clinical trials [ 67]
Tamoxifen	Selective estrogen receptor modulator, nonsteroidal	Competitively binds estrogen receptors; exhibits mixed agonist and antagonist effects	Oral	Anti-tumor effect *in vitro* [66] but increased tumor growth in xenografts [10 ]. Phase II trials showed activity in combination with platinum-based chemotherapy but this was not confirmed in phase III trials [68,69,70]
Toremifene	Selective estrogen receptor modulator, nonsteroidal	Competitively binds estrogen receptors; exhibits mixed agonist and antagonist effects	Oral	Phase II trials showed activity in combination with platinum-based chemotherapy but this was not confirmed in phase III trials [71,72]

Similar results have been observed with vandetanib, an inhibitor of both VEGFR and EGFR, which when added to fulvestrant inhibited tumor growth in xenografts in greater degree compared with either agent alone [61,64].

Several studies show that aromatase inhibitors are also promising as potential lung cancer treatments. For example, an *in vitro* study demonstrated that letrozole (an aromatase inhibitor), decreased cell proliferation in ER expressing cell lines [35] which was confirmed in an independent *in vivo* experiment where treatment with exemestane (another aromatase inhibitor), resulted in significant tumor reduction in human lung tumor xenografts [61]. The results with selective estrogen receptor modulators (SERMs) such as tamoxifen or raloxifene have been inconsistent, with a few studies showing antitumor activity of tamoxifen or raloxifene *in vitro* [35,66] while others report increased lung tumor growth with tamoxifen in xenografts [10]. Earlier preclinical studies have suggested that SERMs could enhance the effectiveness of platinum chemotherapy and overcome platinum resistance by inhibition of the protein kinase C pathway [68,73,74,75,76]. Given this hypothesis, several clinical studies evaluated the efficacy of the addition of tamoxifen or toremifene to platinum-based chemotherapy in lung cancer. Several phase II studies demonstrated that the addition of tamoxifen or toremifene to chemotherapy in advanced NSCLC was well tolerated and resulted in a reasonable efficacy with response rates ranging from 18% to 25% in patients who had been previously treated with a platinum-based therapy [69,70,71,72,77,78]. However, this was not confirmed in subsequent randomized trials. In a randomized phase II trial, the addition of toremifene to platinum-based chemotherapy was compared with chemotherapy alone in patients with advanced NSCLC and no significant difference in response rate or survival was observed [79]. Similarly, a phase III trial of 307 patients with limited stage small cell lung cancer, the addition of high dose tamoxifen to cisplatin, etoposide, and thoracic radiation did not improve the response rate or the overall survival [80]. SERMs have varying agonistic and antagonistic activity against ERs and the exact mechanism of SERMs in lung cancer ER is not clear, thus further preclinical investigation will be necessary before SERMs can be explored as a potential treatment in ER expressing lung cancer.

Despite the antitumor activity of anti-estrogens and aromatase inhibitors observed in preclinical models, there are insufficient data from clinical studies for hormonal therapies to be used in the clinic. Interestingly, there are several published case reports of anti-estrogen use resulting in clinical tumor response in NSCLC patients. A report presents a case of a never-smoking woman who presented with metastatic lung adenocarcinoma, cytokeratin, EGFR and TTF-1 positive by immunohistochemistry, consistent with adenocarcinoma of lung origin. Her tumor was also found to be ER positive although the subtype was not reported. The patient was initially treated with gefitinib resulting in mild disease response and subsequently an aromatase inhibitor was added to gefitinib resulting in complete resolution of metastatic lesions and the response was durable lasting nearly two years [81]. Another case report also describes a never-smoking woman diagnosed with metastatic lung adenocarcinoma with an immunohistochemistry pattern consistent with a lung primary. The patient was initially treated with gefitinib, resulting in a partial response and was subsequently initiated on an estrogen replacement therapy for menopausal symptoms. Interestingly, the patient was found to have disease progression soon after the initiation of hormone replacement therapy (HRT). Because of this finding, the patient’s tumor specimen was evaluated for ER expression which revealed that the tumor did harbor ER expression and subsequent discontinuation of HRT resulted in tumor regression [82]. This is consistent with the findings of the Women’s Health Initiative (WHI) trial in which approximately 16,600 healthy postmenopausal women were randomized to estrogen and progestin combination HRT or placebo with the goal of evaluating the effect of HRT on various clinical outcomes including cardiovascular disease and breast cancer. Unexpectedly, a significant increase in lung cancer mortality was observed in this trial, primarily in NSCLC, although there were too few events in small cell lung cancer to be reliably evaluated [83]. Interestingly, a parallel trial of estrogen alone HRT *versus* placebo did not affect the lung cancer outcome [84], thus increasing interest in better understanding of the progesterone pathway in lung cancer. There have been a number of observational studies investigating the role of HRT in lung cancer and the results have been mixed with reports of both increased and decreased risk as well as of null associations [85,86,87]. The results of these studies are difficult to interpret given the heterogeneity in methodology such as exposure measurement and adjusted covariates. However, the association between HRT and lung cancer became clearer with the results of the WHI study which was a randomized clinical trial. Another population-based study of tumor registry records examined lung cancer incidence and mortality in approximately 6,600 women who received anti-estrogen therapy for breast cancer. This study showed that while lung cancer incidence was not significantly decreased by anti-estrogen therapy, there was a significant decrease in lung cancer mortality which was consistent with the findings of the WHI trial [88]. The above studies indicate that there is likely a subset of ER expressing NSCLC which is sensitive to hormonal influences, whether intratumoral or systemic. While the above case reports do not provide sufficient clinical evidence for the use of anti-estrogen therapy in lung cancer, they suggest that this is a promising therapy which warrants further investigation in the context of clinical trials.

There are ongoing early phase trials which aim to identify this subset and to determine the most appropriate hormonal therapeutic strategy for this patient population. Currently, there is one published report from a phase I trial in which postmenopausal women with advanced NSCLC received a combination therapy of gefitinib and fulvestrant. The primary endpoint was safety of the combination treatment and the study also aimed to explore molecular predictors of therapy. The study enrolled 22 postmenopausal female patients with stage IV or recurrent NSCLC with any prior therapy and participants received daily gefitinib and monthly fulvestrant injections. The results showed that most patients tolerated the combination therapy well, with observed toxicity mostly due to disease progression. Treatment efficacy was not the primary outcome of this trial, but the reported overall response rate was 15% with 12 week progression free survival and 38.5 week overall survival. Interestingly, high ERβ expression in tumor specimens was associated with an overall survival of 65.5 weeks compared with 21 weeks in low ERβ expressing tumors [89]. This study did not select for EGFR mutation, thus the benefit may have been from gefitinib in EGFR mutation positive patients given that higher ERβ expression has been associated with EGFR mutation positive tumors. Therefore this study cannot provide a definitive conclusion regarding the use of anti-estrogens in lung cancer, however, it suggests that ER expression may be able to predict efficacy of hormonal therapies and warrants validation in larger trials.

There are several ongoing phase II clinical studies that are investigating the efficacy of hormonal therapy in lung cancer [67] (Table 4). Three trials aim to evaluate the efficacy of EGFR tyrosine kinase inhibitor and fulvestrant combination therapy in various clinical settings with two of the trials assessing its efficacy as second-line therapy in all NSCLC, while the remaining trial evaluates this strategy in EGFR mutation positive NSCLC patients. The role of fulvestrant is also being evaluated in combination with an aromatase inhibitor as a maintenance strategy after completion of first line chemotherapy in NSCLC patients. Tissue expression of ER is required in only one of these trials and rest of the studies plan to assess ER expression as correlative tissue analysis [67]. There are many challenges associated with designing and conducting of such clinical trials. The first challenge is associated with the development of a reliable diagnostic method in assessment of ER expression and determination of a clinically meaningful definition of positivity. Another challenge stems from the fact that ER expressing NSCLC patient population likely comprises a small proportion of all lung cancer patients thus making timely patient accrual difficult. Additionally, with rapid advances in our understanding of molecular aberrations in lung cancer and ensuing changes in standards of lung cancer therapy, the study design at the initiation of trial may not be relevant at the time of trial completion. Therefore, the success of future clinical trials of hormonal therapy will depend on close collaboration between lab scientists and clinical trialists to develop reliable diagnostic methods and design the most clinically relevant trials. It will also require effective inter-institutional collaborations in conducting these trials to ensure rapid accrual and completion of studies.

**Table 4:** Ongoing phase II clinical trials of hormonal therapy in advanced NSCLC [67].

Patient population	Allowed prior therapy	Preselected biomarker	Treatment	ER/PR assessment	ClinicalTrials.gov Identifier
Stage III or IV NSCLC, postmenopausal women, EGFR mutation positive	0–1 prior chemotherapy	EGFR mutation	Gefitinib + fulvestrant *versus* gefitinib	Not defined	NCT01556191
Stage III or IV NSCLC, postmenopausal women, EGFR wildtype	1–2 prior chemotherapy	EGFR wildtype	Erlotinib + fulvestrant *versus* erlotinib	Not defined	NCT01556191
Stage IIIB or IV NSCLC, both gender, ER or PR positive	Stable disease on erlotinib >2 months, prior chemotherapy not defined	ER and PR expression	Erlotinib + fulvestrant (single arm)	Evaluation prior to trial entry	NCT00592007
Stage IIIB or IV NSCLC, both gender	≥1 prior chemotherapy	None	Erlotinib + fulvestrant *versus* erlotinib	Correlative tissue analysis	NCT00100854
Stage IIIB or IV, postmenopausal women	Completed 6 cycles of first line platinum based chemotherapy	None	Maintenance therapy: (1) Best supportive care *versus* ; (2) Bevacizumab *versus* ; (3) Fulvestrant + anastrozole *versus*; (4) Fulvestrant + anastrazole + bevacizumab	Correlative tissue analysis	NCT00932152

## 5. Conclusions

The current literature provides ample evidence that hormonal pathways play an important role in lung carcinogenesis. The mechanism is likely complex with interplay between estrogen and progesterone receptors, aromatase with intratumoral estradiol, and systemic circulating estrogen and progesterone. This interaction is likely to vary by gender and postmenopausal status in women. Despite this complexity, the discovery of hormonal pathways in lung carcinogenesis provides another layer of depth in our understanding of lung cancer biology and provides a promising opportunity in the development of novel therapeutics employing hormonal strategies which will be possible with close collaborations across disciplines and institutions.
